# Geopolymer Concrete Physical and Mechanical Properties on a Combined Binder Reinforced with Dispersed Polypropylene Fiber

**DOI:** 10.3390/polym17121710

**Published:** 2025-06-19

**Authors:** Sergei A. Stel’makh, Alexey N. Beskopylny, Evgenii M. Shcherban, Diana Elshaeva, Andrei Chernilnik, Denis Kuimov, Alexandr Evtushenko, Samson Oganesyan

**Affiliations:** 1 Department of Unique Buildings and Constructions Engineering, Don State Technical University, 344003 Rostov-on-Don, Russia; sergej.stelmax@mail.ru (S.A.S.); diana.elshaeva@yandex.ru (D.E.); chernila_a@mail.ru (A.C.); aevtushenko@donstu.ru (A.E.); alfateh@list.ru (S.O.); 2 Department of Transport Systems, Faculty of Roads and Transport Systems, Don State Technical University, 344003 Rostov-on-Don, Russia; 3 Department of Engineering Geometry and Computer Graphics, Don State Technical University, 344003 Rostov-on-Don, Russia; au-geen@mail.ru; 4 Department of Digital Technologies and Platforms in Electric Power Industry, Faculty of Energy and Oil and Gas Industry, Don State Technical University, 344003 Rostov-on-Don, Russia; d.kuimov@sci.donstu.ru

**Keywords:** aluminosilicate component, geopolymer concrete, compressive strength, dispersed reinforcement, polypropylene fiber (PF)

## Abstract

Geopolymer concrete is a promising construction material that acts as an alternative to cement concrete. Unlike traditional cement concrete, geopolymers are environmentally friendly materials, the production of which does not involve significant carbon dioxide emissions. However, the structure formation and properties of geopolymers significantly depend on raw materials and are insufficiently studied. The aim of the study is to select the optimal combination of ground granulated blast furnace slag (GGBS) and fly ash (FA) as a binder and the optimal content of polypropylene fiber to create a sustainable, environmentally friendly and effective geopolymer concrete. To study various compositions of geopolymer binders selected by combining GGBS and FA, experimental geopolymer concrete mixtures and samples from them were manufactured. The density and slump of fresh concrete and the density and compressive strength of hardened composites were studied as mechanical characteristics. The microstructure of the geopolymer matrix was analyzed using optical and scanning electron microscopes. The most rational combination of GGBS 80% and FA 20% was determined, which allows obtaining a composite with the highest compressive strength of up to 31.5 MPa. A dispersion reinforcement study revealed that 0.8% polypropylene fiber (PF) is optimal. This allowed us to increase the compressive strength by 7.3% and the flexural strength by 48.7%. The geopolymer fiber concrete obtained in this study is a sustainable and environmentally friendly alternative composite material and has sufficient performance properties for its use as an alternative to cement concrete.

## 1. Introduction

The global demand for sustainable technological solutions in the construction industry is rapidly growing. The key and most popular building material in the field of civil and industrial construction is cement-based concrete [1]. Cement production is accompanied by emissions of a huge amount of greenhouse gases—up to 7% of global emissions [2]. The development of alternative environmentally friendly materials that can replace cement concrete is currently a relevant area of study. Geopolymer concrete is a building material with a reduced impact on the environment, comparable in its characteristics to cement concrete [3,4,5].

In the current literature, alkali-activated aluminosilicate binders are considered a promising replacement for Portland cement. The main advantages of these binders include the fact that the process of their preparation is quite economical and does not require calcination, firing and direct extraction of natural resources and their processing. The main raw material for their production is waste from the industrial and fuel and energy complex. This type of binder is also highly environmentally friendly and will reduce the carbon footprint [6,7]. However, despite the huge economic potential and environmental friendliness of aluminosilicate binders, there are several limitations that hinder their widespread use. First, this is the complexity of developing a binder formulation of optimal composition and the instability of the chemical composition of aluminosilicate components. Thus, the search for new formulation solutions and the development of optimal compositions of geopolymer binders for obtaining geopolymer composites with improved performance properties are relevant and are highly popular in the modern global materials science community [8,9,10].

The most popular aluminosilicate components for obtaining geopolymer binders are fly ash (FA), ground granulated blast furnace slag (GGBS) and other types of slag. Geopolymer mixtures on GGBS with a compressive strength of up to 62.6 MPa have already been developed [11]. In addition, a geopolymer composite based on GGBS with the addition of gypsum and NaCl has already been obtained, which significantly increased its compressive strength up to 82.3% and resistance to sulfide corrosion [12]. A combination of copper slag and 2% microsilica allowed us to obtain a geopolymer with a compressive strength of 79 MPa [13]. A combination of GGBS, rice husk ash and lightweight clay filler allowed us to obtain lightweight geopolymers with a density of up to 1868 kg/m^3^ and a compressive strength of up to 37.1 MPa [14]. The efficiency of using GGBS as a base for a geopolymer binder is also confirmed by several other studies [15,16,17,18]. Another popular aluminosilicate component is ash of various origins, including fly ash (FA). Previously, it was possible to obtain stable geopolymer concrete on FA with the addition of microsilica [19]. Ash from the incineration of solid municipal waste in combination with an alkaline activator of optimal composition exhibits the properties of a binder and allows obtaining a composite with a compressive strength of 43.7 MPa [20]. Correctly selected geopolymer composites on FA are actively used in 3D printing technology [21]. Similar experimental results confirming the effectiveness of FA in geopolymer concrete technology are reflected in several other studies [22,23,24,25,26,27]. Using multiple aluminosilicate components, including GGBS, in optimal combinations enables the creation of geopolymer composites with desired performance characteristics [28,29,30,31,32,33].

In general, most of the studies devoted to geopolymer composites are aimed at developing the optimal composition of the geopolymer binder and selecting the best composition of the alkali activator for it [34,35]. Using fiber to improve the properties of various types of composites is a popular formulation solution and is actively studied as one of the options for improving the strength properties of geopolymer concrete [36]. For example, the addition of steel fibers to geopolymer concrete made it possible to increase its compressive strength by 23.2% [37]. Basalt fibers show good results in the geopolymer’s structure, limiting its shrinkage cracks and improving strength properties [38,39]. Including steel fiber and polyvinyl fibers made it possible to increase the strength properties of the geopolymer when exposed to a temperature of 200 °C [40]. The study of the behavior of geopolymer concrete under the influence of high temperatures proves its competitiveness in comparison with cement composites in this indicator [41,42].

Polypropylene fibers, introduced into the composition of the geopolymer composite in an amount of 1%, also positively affect its properties, increasing the compressive strength by up to 18% [43]. Improving the mechanical properties and structure of geopolymer composites by adding polypropylene fibers has been proven previously [44]. The inclusion of 0.8% polypropylene fibers in the composition of geopolymer concrete for 3D printing has increased its strength properties and improved plasticity [45]. Also, the addition of polypropylene fibers improves the performance characteristics of geopolymer concrete, counteracting the penetration of chlorides [46]. The positive effect of dispersed reinforcement of geopolymer composites with various types of fibers has been proven in several other studies [47,48,49]. The development of new types of sustainable geopolymer concretes using various types of man-made waste is highly relevant, especially for those regions where there is a problem of industrial waste accumulation. It is important to note that the development of several types of waste combinations in the geopolymer composition is the most desirable result. However, the development of such compositions is quite complex and requires a large amount of experimental research aimed at studying the properties of the waste itself and the properties of finished composites. To achieve geopolymer concrete with the required operational properties based on waste, it is necessary to search for many various formulations and technological solutions, which significantly limit the geopolymer concrete usage in construction practice. Thus, based on the results of the review, a deficit of works was established in which the effect of polypropylene fiber on the structure formation and characteristics of geopolymer concrete on a combined binder was studied [11,25]. The main gaps in the research are in studying the effect of reinforcement of geopolymer concrete with polypropylene fiber on a combined binder and obtaining the dependencies of the properties of such concrete on the parameters of its composition from precursors of local origin.

Thus, the scientific novelty of the work lies in studying the structure and obtaining new dependencies of the properties of geopolymer concrete on a combined binder based on GGBS and FA and dispersed-reinforced with polypropylene fiber of local origin on the parameters of the composition.

The objective of this study is to select the optimal combination of ground blast-furnace granulated slag and fly ash as a binder and the optimal content of polypropylene fiber to create a sustainable, environmentally friendly and effective geopolymer concrete. Based on this, the following tasks were formulated:

− Development of experimental compositions of geopolymer concrete on a combined binder with different ratios of GGBS and FA and selection of the optimal dosage of polypropylene fiber;
− Production of fresh geopolymer concrete on a combined binder and control of its density and slump;
− Production of geopolymer concrete samples and evaluation of its density and compressive and flexural strength. Analysis of the structure of geopolymer composite reinforced with polypropylene fiber;
− Analysis of the experimental results and selection of the most optimal formulation of geopolymer fiber-reinforced concrete, allowing us to obtain a composite with the required performance properties;
− Development of recommendations for the practical use of the obtained geopolymer concrete reinforced with dispersed polypropylene fiber.

## 2. Materials and Methods

### 2.1. Materials

**Figure 1:**
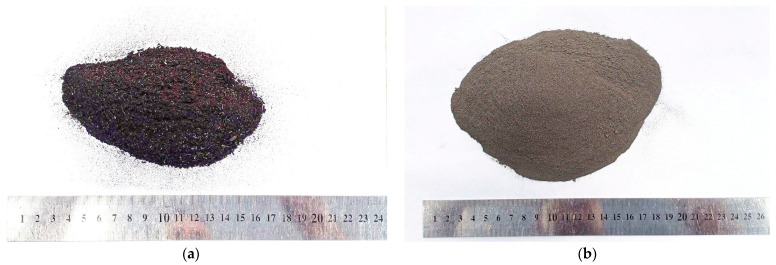
Appearance of aluminosilicate components: (**a**) FA, (**b**) GGBS.

Ground blast furnace granulated slag (GGBS) (Severstal, Cherepovets, Russia) and fly ash (FA) (Novocherkassk GRES, Novocherkassk, Russia) were used as the major components for the aluminosilicate binder. The chemical composition of GGBS and FA and their appearance are presented in Table 1 and Figure 1, respectively. The chemical composition of FA and GGBS was provided by suppliers.

Quartz sand (QS) (Nedra, Samarskoye village, Russia) was used as a fine aggregate with the following characteristics: bulk density—1402 kg/m^3^; apparent density—2594 kg/m^3^; the content of dust and clay particles—0.03%; content of clay in lumps—0.05%; no organic and contaminant content.

Crushed granite (CrS) (Pavlovsk Nerud, Voronezh, Russia) was used as a coarse aggregate with the following characteristics: bulk density—1435 kg/m^3^; apparent density—2663 kg/m^3^; resistance to fragmentation—11.0 wt %; content of plate-like and needle-like grains—5.2 wt %.

**Figure 2:**
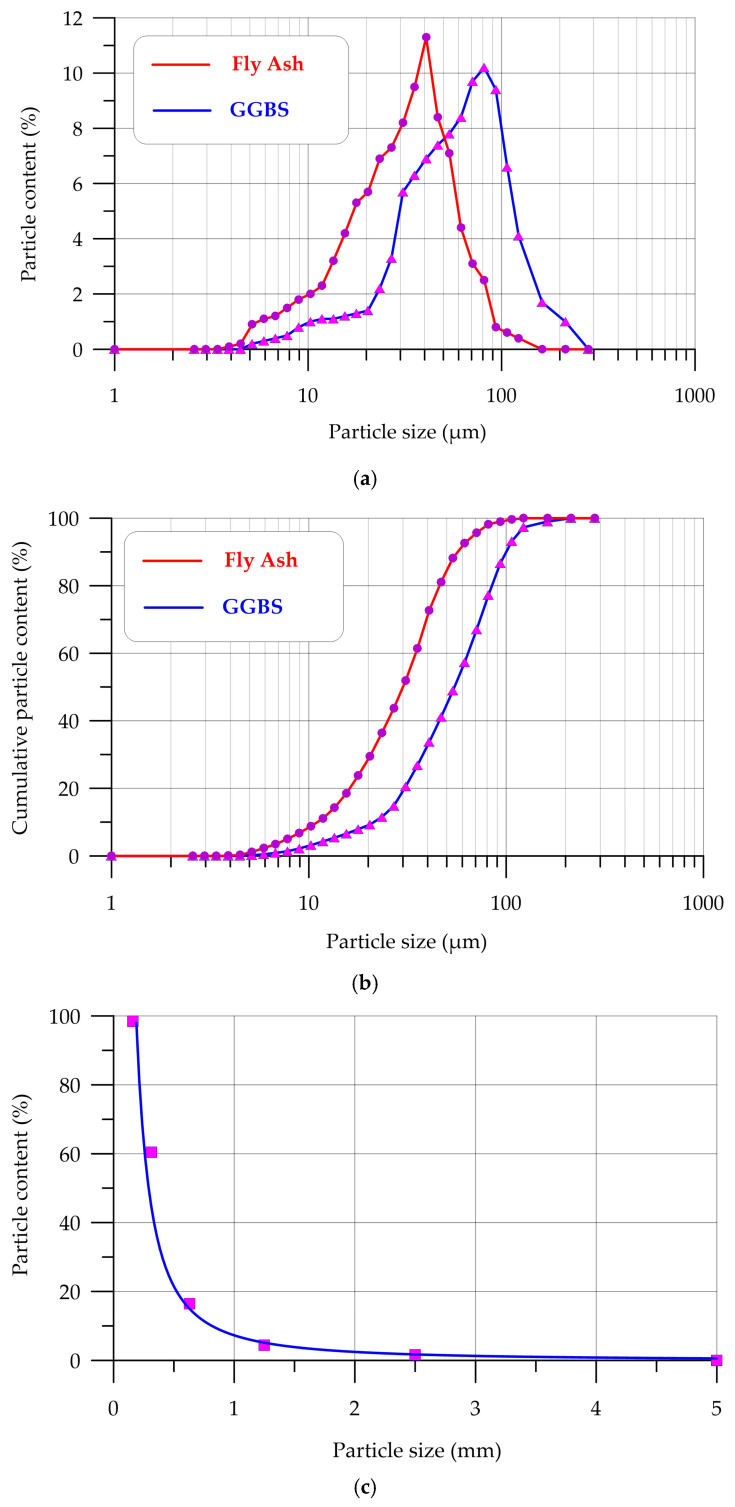

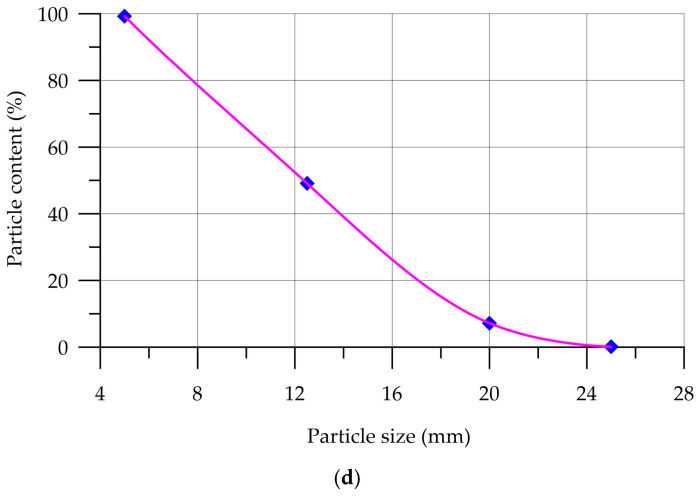
Particle distribution curves of (**a**) GGBS and FA; (**b**) GGBS and FA cumulative content; (**c**) QS; (**d**) CrS.

The distribution curves of GGBS, FA, QS, CrS particles are shown in Figure 2.

Based on the analysis of the curves in Figure 2, it was established that the main share of GGBS particles, 82.5%, ranges in size from 30 to 150 μm. The majority (81.4%) of FA particles are sized between 10 μm and 60 μm. Fine filler QS has a fineness modulus of 1.82. Coarse filler has a grain size of 5 to 20 mm.

The particle size affects the density of the composite structure during the geopolymerization reactions and the water requirement of the mixture, which affects the characteristics of geopolymer concrete, so it is important to select such granulometric compositions of the components that will ensure dense packing of particles by filling intergranular voids, and without additionally increasing the water requirement of the mixture.

Sodium hydroxide (NaOH) (Khimprom, Novocheboksarsk, Russia) was used as an alkaline activator.

Polypropylene fiber (Armplast, Taganrog, Russia) with the following characteristics was used for dispersed reinforcement: fiber diameter—12 μm; fiber length—12–16 mm; tensile strength—320 MPa; density—0.91 g/cm^3^.

### 2.2. Methods

Development of the optimal composition of geopolymer fiber concrete on a combined binder included two main stages. First, the optimal ratio of GGBS and FA in the concrete composition was selected. Second, the optimal percentage of reinforcement of the PF geopolymer composite was selected. In the first stage, 6 compositions of geopolymer concrete were developed (Table 2). The percentage ratio of GGBS and FA varied with an interval of 20%. The optimal amount of NaOH and water for preparing the alkaline activator was determined by calculation during the selection of the composition of geopolymer concrete.

**Figure 3:**
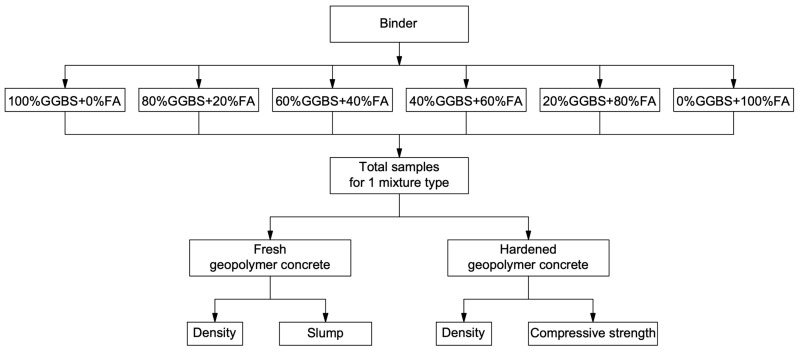
Experimental study program of geopolymer concrete on a combined binder.

The program of the first stage of experimental research is presented in Figure 3.

**Figure 4:**
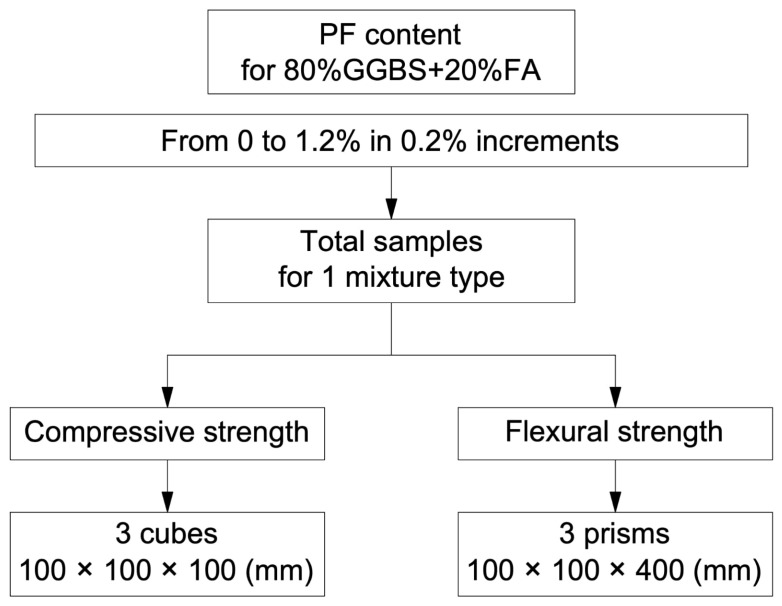
Experimental study program for geopolymer fiber-reinforced concrete with combined binder.

In the second stage of experimental studies, 6 compositions of geopolymer concrete with different PF content were developed. The formulation of geopolymer fiber concrete and the experimental study program are presented in Table 3 and Figure 4, respectively.

The study adopted a range of fiber dosages from 0.2 to 1.2% due to the analysis of the previously published literature concerning the use of polypropylene fiber in geopolymer concrete and preliminary test experiments conducted by the authors earlier, which revealed the inappropriateness and irrationality of expanding the range, due to a noticeable deterioration in the performance of the finished composite [18,25,45,46,50,51].

Preparation of fresh geopolymer concrete and molding of experimental samples both at the first and second stages of the experimental study was carried out in a similar manner and included the following main stages: dosing of all raw materials according to the recipe; preparation of an alkaline solution (an alkaline solution with a NaOH concentration of 14.18% was prepared 24 h before use); GGBS, FA and QS were poured into a mixer and mixed dry for 60 s; then the alkaline solution was poured in and everything was mixed until smooth; CrS was added to the finished geopolymer mixture and was mixed until smooth. PF was added at the very end, and the mixture was additionally mixed; the finished geopolymer concrete mixture was unloaded from the mixer and poured into cube molds and prism molds in 3 stages; at each stage, the mixture was additionally compacted using a bayonet; then the filled forms were placed on a laboratory vibration platform SMZh (Imash, Armavir, Russia) and compacted for 60 s; after compaction, the surface of the samples was smoothed, and they were kept in the molds for 10 h, after which they were subjected to heat and moisture treatment (steaming was carried out for 10 h, the temperature of isothermal heating was 85–90 °C); after heat and moisture treatment, the samples were cooled and removed from the molds; then they were placed in a normal hardening chamber until they reached the age of 28 days. The curing conditions in such a chamber were as follows: temperature 20 ± 2 °C, relative humidity 95 ± 5%.

Unlike Portland cement concrete, the hardening rate of geopolymer concrete largely depends on the composition of the grout (alkaline activator), and with a correctly selected composition of the alkali activator, the presence of additives that accelerate hardening and heat treatment, the geopolymer can sometimes gain design strength faster than Portland cement concretes. However, in most current studies devoted to slag-based geopolymer concretes, the optimal hardening time for them, like Portland cement concretes, is considered to be 28 days [52,53].

This mode of heat and moisture treatment was chosen after analyzing previously obtained data [54,55,56], where the efficiency of heat and moisture treatment of geopolymer binders and concretes based on them was explained by the fact that at high curing temperatures, Ca, Al and Si react more quickly with water and form a high-density CASH gel.

Polypropylene fiber was introduced into the finished geopolymer concrete mixture. The fibers were divided into approximately 3 equal parts; each part was evenly scattered over the entire surface of the mixture and mixed for 30 s. This operation was repeated and ensured the most uniform distribution of fiber throughout the entire volume of the mixture. The homogeneity of the fiber dispersion was checked on fresh geopolymer concrete samples and hardened specimens after strength tests, both visually and using an optical microscope.

Preliminary holding in molds and steaming were conducted in order to intensify the rate of geopolymer reactions. GGBS and FA are pozzolanic active mineral substances, and as is known, their hardening rate is slower in comparison with Portland cement [57].

The slump of the cone and the density of freshly made concrete mixtures were determined in accordance with the method requirements [58]. To determine the slump, a cone, a metal sheet and a ram, previously wiped with a damp cloth, were used. The cone was placed on the sheet and filled with the mixture in 3 stages. Each layer was compacted with 25 blows of the ram. After compacting the mixture, its excess was cut off with a ruler. The cone was raised up and placed nearby. Then the slump of fresh geopolymer concrete was measured. The density, compressive strength and flexural strength of geopolymer concrete and fiber concrete were determined according to the methods [59,60,61,62,63,64,65]. Cube samples were first used to determine the density and then to determine the compressive strength. First, their geometric sizes and weights were measured. Then the samples were installed in the Press P-50 laboratory unit (IMASH, Armavir, Russia). The load was applied at a rate of increase of 0.6 ± 0.2 MPa/s. Before testing, the geometric parameters of the prism samples were measured and then installed in a special laboratory setup and loaded at a rate of increase of 0.05 ± 0.01 MPa/s. The results obtained during the tests were processed using the formulas presented in Table 4.

In total, 18 cube samples were made during the first stage of experimental studies to determine the efficiency of the newly developed geopolymer concrete compositions in terms of density and compressive strength. In the second stage of experimental studies, 36 samples were made, including 18 cube samples and 18 prism samples, to evaluate the efficiency of PF dispersed reinforcement. Granulometric analysis of aluminosilicate components and fillers was performed using a Microsizer 201C laser particle analyzer (VA INSALT, St. Petersburg, Russia) and laboratory sieves. The structure of geopolymer concrete and fiber concrete was studied using an MBS-10 optical microscope (Izmeritelnaya Tekhnika, Moscow, Russia) at a magnification of 10 times. The microstructure of the geopolymer matrix was studied using a Tescan scanning electron microscope (Brno, Czech Republic) with an Inca Energy 450/XT electron probe microanalysis system. The images were obtained in backscattered electrons (BEs) or secondary electrons (SEs). Areas with a higher average atomic weight (relatively enriched in heavy elements) are colored in lighter shades. In BEs, areas with a higher average atomic weight (relatively enriched in heavy elements) are colored in lighter shades.

## 3. Results and Discussion

### 3.1. The Performance of Geopolymer Concrete Evaluation on a Combined Binder

**Figure 5:**
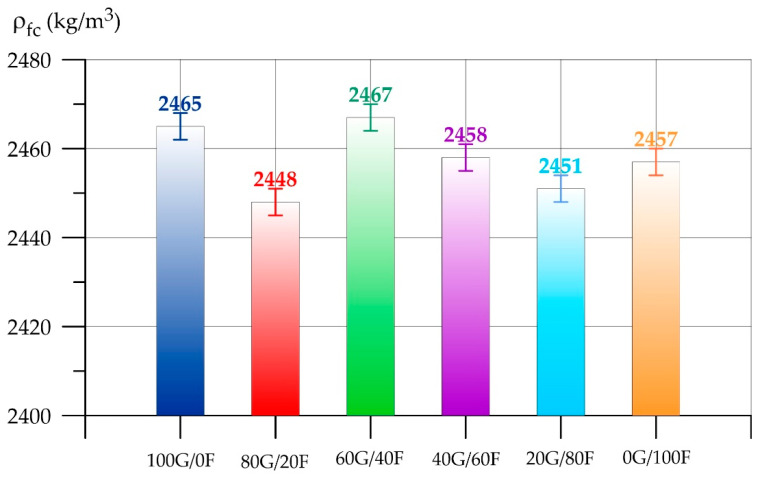
Density of fresh geopolymer concrete as a function of GGBS and FA content.

**Figure 6:**
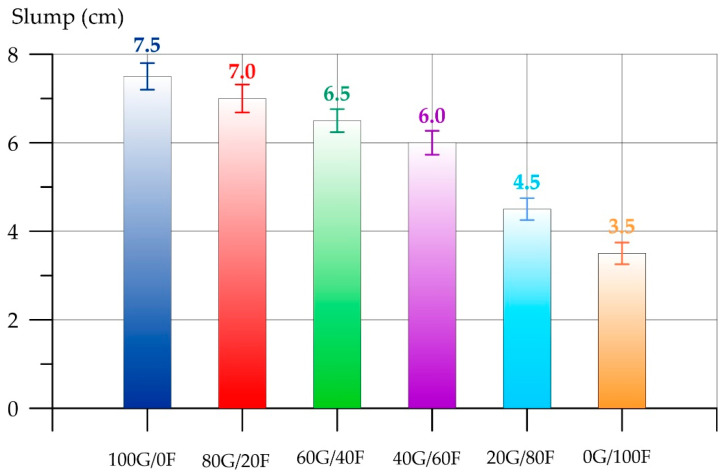
Dependence of fresh geopolymer concrete slump on GGBS and FA content.

Figure 5 and Figure 6 show the results of determining the density ($\rho_{fc}$) and the slump of fresh geopolymer concrete, respectively.

According to the data on determining the density of fresh geopolymer concrete (Figure 5), a change in the content of aluminosilicate components GGBS and FA in the binder does not significantly affect the density of the geopolymer concrete mix. Deviations from the control composition of type 100G/0F varied from 0.1% to –0.7%.

**Figure 7:**
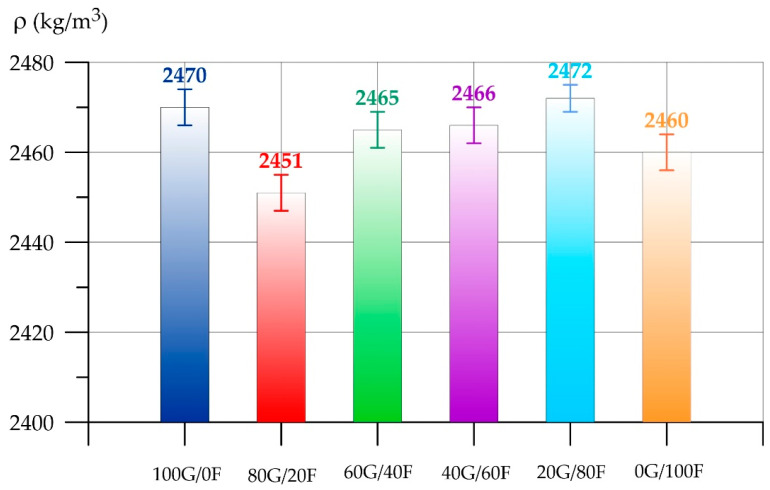
Dependence of the density of geopolymer concrete on the content of GGBS and FA.

**Figure 8:**
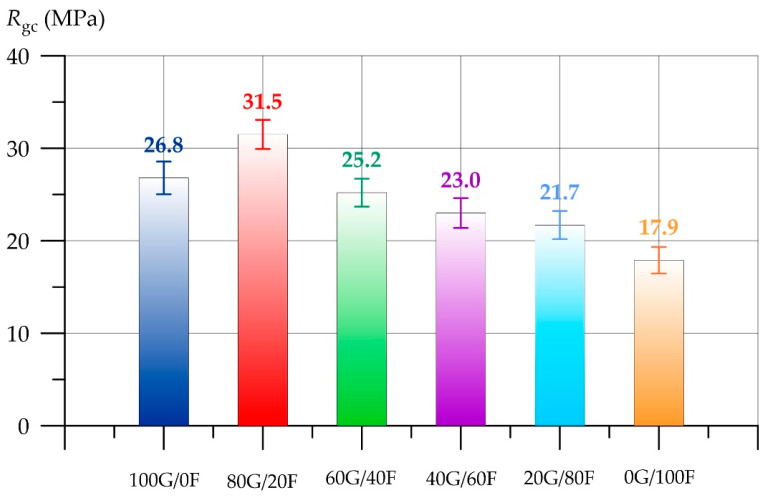
Compressive strength of geopolymer concrete as a function of GGBS and FA content.

As can be seen from Figure 6, the highest value of fresh geopolymer concrete slump was recorded for the composition of type 100G/0F, where GGBS was the main component of the binder. The lowest slump value is for the composition of type 0G/100F. The slump of the 0G/100F composition on the FA binder is 53.3% less than that of the 100G/0F composition. With an increase in the proportion of FA in the combined binder, the slump in the mixtures decreases. For the 80G/20F, 60G/40F, 40G/60F and 20G/80F type compositions, the slump values decreased by 6.7%, 13.3%, 20.0% and 40.0%, respectively. The decrease in the slump of the geopolymer concrete mixture with an increase in the FA content is explained by the fact that FA particles have a higher water demand compared to GGBS due to their smaller size. Based on the analysis of the particle distribution curves, it was found that the main share of GGBS particles varies in the size range from 30 to 150 μm, and FA particles in the range from 10 μm to 60 μm. More alkaline activators will be spent on wetting the FA particles, which they absorb from themselves from the total volume of the mixture, thereby reducing their workability. For 20G80F and 0G100F, the drop becomes more abrupt due to the fact that the proportion of FA increases by four or more times compared to GGBS, which leads to a sharp increase in the water demand of the mixture due to the smaller size of FA particles compared to GGBS. Much more alkaline activator will be spent on wetting the FA particles, which they absorb from the total volume of the mixture, thereby significantly reducing the sediment of the mixture [66,67]. Further, Figure 7 and Figure 8 present the results of determining the density (*ρ*) and compressive strength (*R*_gc_) of geopolymer concrete, respectively.

As with the density of fresh geopolymer concrete, the density of the hardened composite changed insignificantly. Deviations from the 100G/0F composition ranged from 0.1% to –0.8%. Changing the ratio of GGBS and FA in the geopolymer binder does not significantly affect the density of the concrete composite since the bulk density of the applied GGBS and FA differs insignificantly in dry form.

**Figure 9:**
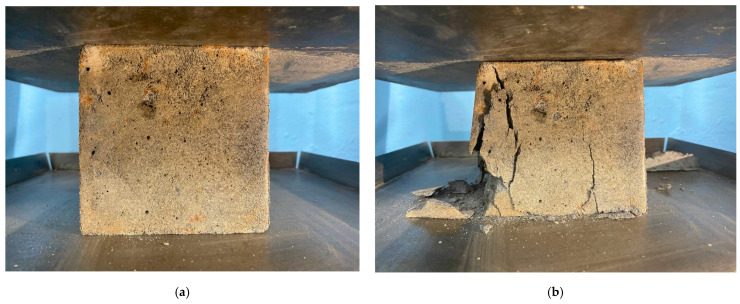
A sample of geopolymer concrete of the 80G/20F type (**a**) before failure; (**b**) after failure.

Based on the results of determining the compressive strength, shown in Figure 8, it was found that geopolymer concrete on a combined binder consisting of 80% GGBS and 20% FA has the highest strength. The compressive strength of the 80G/20F composition was 17.5% higher than that of the 100G/0F composition. The combination of 80% GGBS and 20% FA allows us to obtain a geopolymer binder system with the most rational combination of Ca/Si. It is the content of these elements that is responsible for the activity of geopolymerization reactions and the amount of formed hydrosilicate gel (CSH), which binds all raw materials and directly affects the strength of geopolymer concrete. Further, as the proportion of GGBS decreases and the proportion of FA increases, a negative effect is observed, which is associated with a decrease in the amount of formed CSH [68,69,70]. Further, as the proportion of GGBS decreased and the proportion of FA increased, a negative effect was observed. The compressive strength of the 60G/40F, 40G/60F, 20G/80F and 0G/100F compositions was lower by 6.0%, 14.2%, 19.0% and 33.2%, respectively. The appearance and nature of the destruction of geopolymer concrete of the 80G/20F type composition are shown in Figure 9.

The sample of geopolymer concrete shown in Figure 9 has a yellow-gray tint. Failure occurred mainly in the central and left side parts of the sample.

The reason that the 80G/20F composition has the highest compressive strength is that this geopolymer system has a denser hydrosilicate gel (CSH) with a high Ca/Si content, which actively fills all the voids at the interface of all raw materials. Accordingly, geopolymer systems with lower compressive strength have less dense CSHs and a low Ca/Si content. An increase in the FA content in this system reduces the reactivity. Also, a number of other studies have shown that alkali-activated pastes based on GGBS, as a rule and in most studies, have higher strength than alkali-activated FA pastes based on fly ash [68,69].

The obtained result in the selection of the optimal composition of the geopolymer combined binder is consistent with many other studies, where the authors also managed to develop effective and environmentally friendly geopolymer composites by combining various aluminosilicate components in the binder (Table 5).

Combining GGBS and FA allowed us to select a geopolymer binder with the optimal composition. With a combination of 80% GGBS and 20% FA, geopolymerization reactions proceed most intensively, and a greater amount of CSH is formed, which directly affects the final strength of the composite. The 80G/20F composition was accepted as the most effective and was taken as a basis for further study of the effect of dispersed reinforcement with polypropylene fiber.

### 3.2. Assessment of Geopolymer Concrete Performance on a Combined Binder Reinforced with Polypropylene Fiber

**Figure 10:**
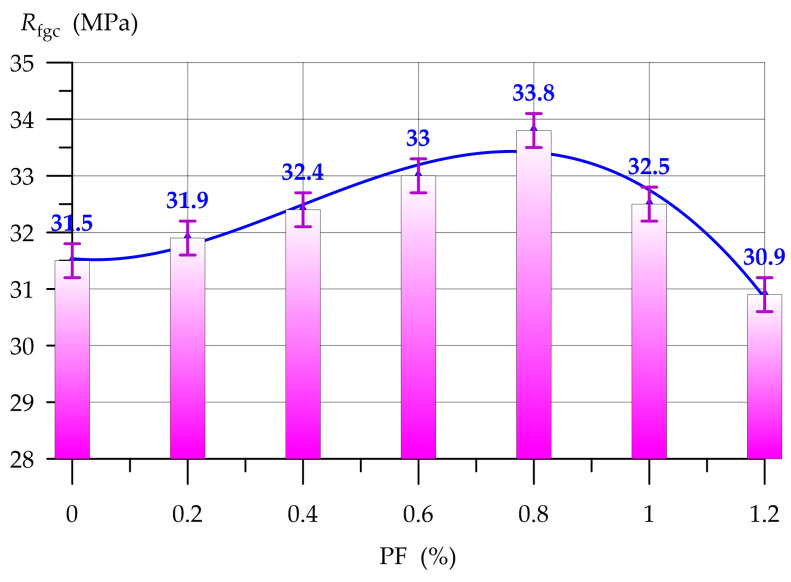
Compressive strength of geopolymer concrete as a function of PF content.

**Figure 11:**
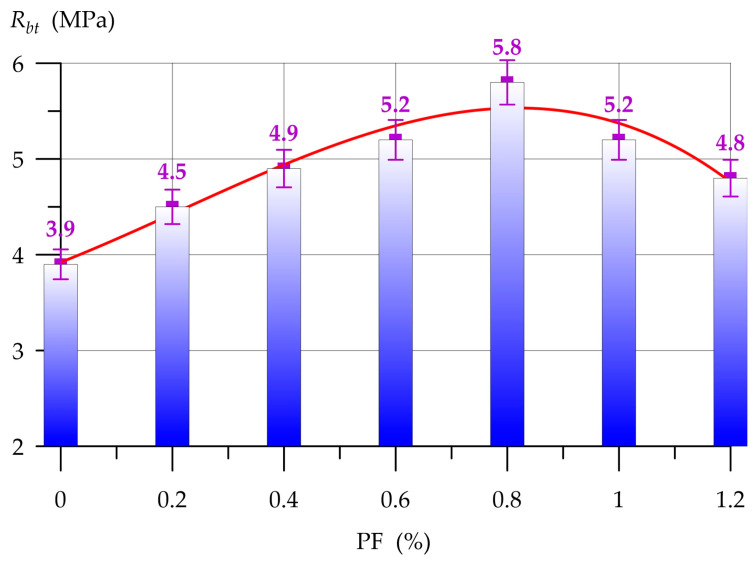
Geopolymer concrete flexural strength versus PF content.

Figure 10 and Figure 11, respectively, show the results of determining the compressive strength $R_{fgc}$ and flexural strength $R_{bt}$ of geopolymer concrete with PF.

The dependencies presented in Figure 10 and Figure 11 are well approximated by polynomial functions of degree 4 and 3, respectively:

(5) $$R_{fgc}=31.53-1.011\;x+13.23\;x^{2}-12.44\;x^{3}+1.42\;x^{4},\;\;\;\;\;\;R^{2}=0.95$$

(6) $$R_{bt}=3.92+2.305\;x+1.577\;x^{2}-2.430\;x^{3},\;\;\;\;\;\;R^{2}=0.938$$

here *x* is PF content, %; *R*^2^ is the coefficient of determination.

Figure 10 shows that the introduction of PF into the concrete composition has a positive effect on the strength of the geopolymer composite. With a PF content of 0.2% to 0.8%, a stable increase in compressive strength is observed. The maximum compressive strength was recorded at 0.8% PF, the increase was 7.3%. Then, with a content of 1.0% and 1.2% PF, an inverse relationship is observed: the efficiency of dispersed PF reinforcement decreases. With 1.0% PF, the compressive strength is 3.2% higher than the control composition, and with 1.2% PF, the compressive strength is 1.9% lower.

As can be seen from Figure 11, geopolymer concrete with PF from 0.2% to 0.8% has a stable trend to increase flexural strength. The maximum value of the increase in bending strength was recorded at 0.8% PF and amounted to 48.7% compared to the control composition. The efficiency of dispersed reinforcement decreases for higher PF contents—1.0% and 1.2%. The increases in bending strength were 33.3% and 23.1%, respectively.

The decrease in the efficiency of dispersed reinforcement with the introduction of 1.0% or more PF occurs due to the supersaturation of geopolymer concrete with fiber. Too high dosages of dispersed reinforcing fiber lead to the fact that, at the manufacturing stage, these fibers cannot be evenly distributed throughout the entire volume of the composite and clump. In areas where such formations are present, there will be reductions in strength. The presence of such zones negatively affects the properties of the composite, and the effect of dispersed reinforcement is reduced or even leads to deterioration of the properties of geopolymer composites [35,75].

Thus, based on the results of determining the compressive and bending strength of geopolymer concrete on a combined binder, it was found that dispersed PF reinforcement has a positive effect on these properties. The introduction of PF into the composition of geopolymer concrete up to 1% has a positive effect on compressive and bending strength. At higher dosages, an inverse relationship is observed in the form of a decrease in strength properties. The PF content of 0.8% is optimal for this type of geopolymer concrete. PF, introduced into the geopolymer composite in an optimal amount, is distributed uniformly throughout its volume and promotes the creation of macrostructural cells, where fiber fibers additionally bind together the binding components, sand and crushed stone. Due to the formation of these macrostructural cells, dispersed-reinforced geopolymer concretes better resist the effects of destructive loads. However, discrepancies arise in the operational mechanisms of PF within the composite structure when evaluating compressive and flexural strengths. The mechanism of PF operation under compressive load can be described as follows. Fibers have a chaotic distribution in the composite body, part of the stresses from the compressive load are perceived by themselves and additionally hold the structure of the composite in all directions [76]. Under the action of a bending load, fibers also perceive part of the stresses and resist it until complete destruction. It is this difference in the features of the fiber operation mechanism that explains the higher increases in bending strength compared to compressive strength. The PF content above the norm leads to a negative effect. The fibers cannot be distributed normally and evenly throughout the entire volume of the composite. They clump together, forming zones of low strength, which lead to a weakening of the entire structure of the geopolymer composite. In general, the use of various types of fiber in geopolymer composite technology is a popular formulation solution [77,78]. The results obtained in this section of the study are in good agreement with the works of other authors (Table 6).

Dispersed reinforcement of the PF geopolymer composite in the amount of 0.8% of the combined binder mass is a rational formulation solution and allows obtaining geopolymer concrete with additionally improved strength properties.

**Figure 12:**
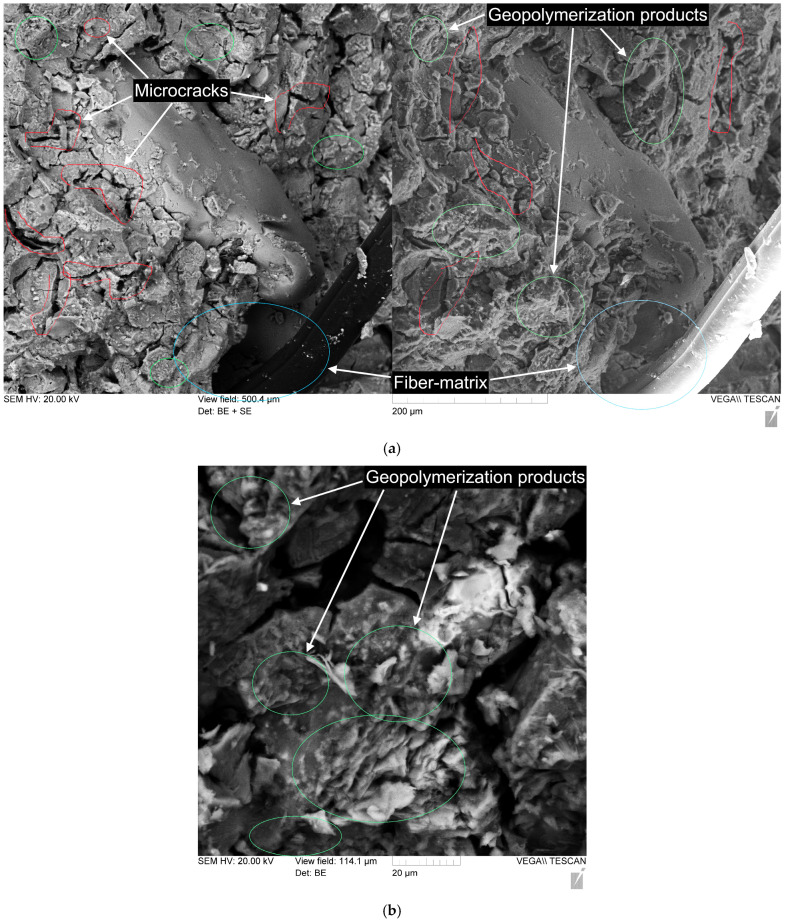
Microstructure of geopolymer paste of composition 80G/20F: (**a**) with magnification of 300×; (**b**) with magnification of 1000×.

**Figure 13:**
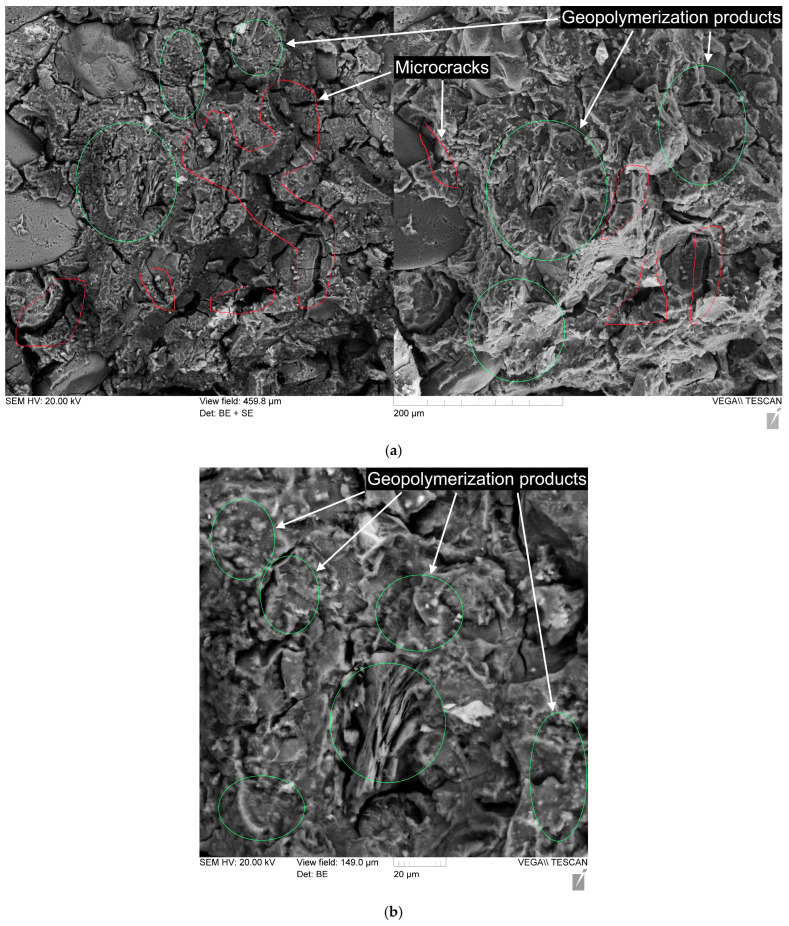
Microstructure of geopolymer paste of composition 80G/20F with 0.8% PF: (**a**) with magnification of 300×; (**b**) with magnification of 1000×.

Figure 12 and Figure 13 show the microstructure of geopolymer paste of composition 80G/20F without PF and with 0.8% PF.

The geopolymer matrix based on the mixture of 80% GGBS and 20% FA has a well-compacted microstructure (Figure 12b and Figure 13b), and a large amount of gel-like products of the geopolymerization reaction is observed. No unreacted GGBS and FA particles are recorded in Figure 12 and Figure 13. It is noted that the geopolymer matrix samples studied after the action of compressive destructive loads have characteristic microcracking (Figure 12a and Figure 13a), represented by thin and short microcracks. The PF at the fiber–matrix interface is pulled out of the matrix structure and deformed under the action of destructive loads. The nature of the destruction at the fiber–matrix interface indicates a sufficiently good adhesion of the matrix and fiber.

**Figure 14:**
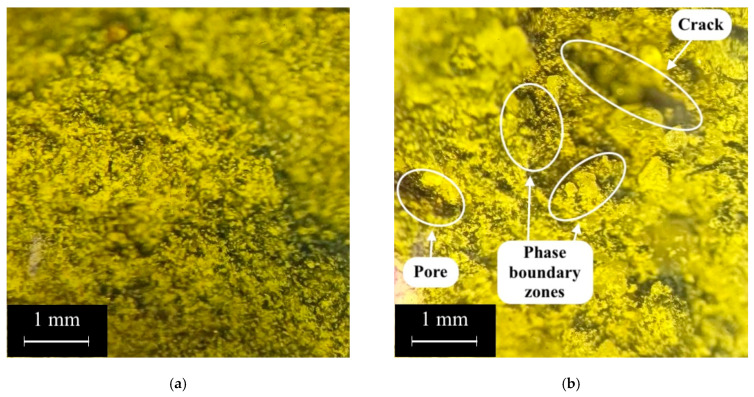
Structure of a sample of geopolymer concrete of type 80G/20F: (**a**) without marking; (**b**) with marking.

**Figure 15:**
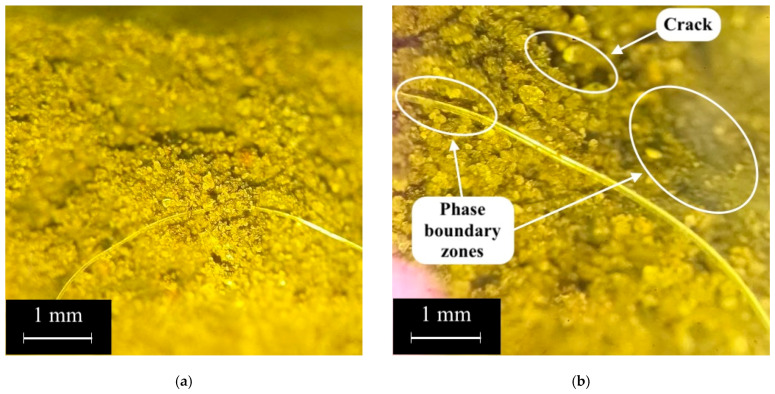
Structure of the sample of geopolymer concrete of the 80G/20F type with 0.8% PF: (**a**) without marking; (**b**) with marking.

Further, in Figure 14 and Figure 15, an analysis of the structure of the geopolymer composite of the 80G/20F type without PF and with 0.8% PF is presented. The photographs were taken at 10× magnification.

The geopolymer concretes shown in Figure 14 and Figure 15 have a fairly uniform structure. The contact zone between the fillers and the geopolymer binder is visually dense, indicating a well-organized structure without noticeable defects (Figure 14b). The PFs are distributed uniformly and are partially extended from the geopolymer matrix, which indicates the standard nature of destruction and deformation of fibers under compressive and bending loads (Figure 15) [79,90,91]. Figure 15 shows the contact zone at the phase boundary between the fiber and the geopolymer binder, and the microcracks that formed in the composite structure during its destruction. The combination of GGBS and FA in optimal quantities and their different granulometric compositions, where for GGBS the main share of particles falls in the range from 30 to 150 μm, and for FA from 10 μm to 60 μm, made it possible to obtain the densest and strongest structure. FA particles are smaller than GGBS particles, and due to this, they can fill the voids between them, and thus, a denser composite structure is created during the geopolymerization reactions [72,91,92,93]. Thus, the conducted study of the structure confirms the obtained dependencies of the characteristics of geopolymer concrete on a combined binder based on GGBS and FA, dispersed-reinforced with polypropylene fiber, on the parameters of its composition.

The results of this experimental study reflect the following specific results:

- Qualitative and quantitative characteristics of the feedstock for its further use in geopolymer concrete technology were determined;
- The most rational compositions of the geopolymer binder system for obtaining highly effective geopolymer concretes were obtained;
- The optimal composition of the geopolymer binder was selected by combining 80% GGBS and 20% FA with a compressive strength of 31.5 MPa;
- The optimal PF content of up to 0.6–1.0% was selected for dispersed reinforcement of geopolymer concrete.

The obtained results develop and complement the theoretical and experimental dependencies of the global science of geopolymer composites [51,55,81]. The obtained geopolymer concrete has sufficient strength properties for its extensive use in real construction practice. The developed geopolymer fiber concrete can be actively used both for the manufacture of various factory-made products and in construction site conditions. The main advantages of this geopolymer fiber concrete include its high environmental friendliness, low carbon impact on the environment and cost-effectiveness. Using slag and ash for manufacturing concrete simultaneously allows for solving several problems. The problem of recycling industrial and fuel and energy complex waste is solved, the rejection of cement will significantly reduce the cost of concrete production and, at the same time, solve the problem of reducing the carbon impact [93,94,95]. However, it is worth noting the limitations of this study, which are primarily associated with the features of the aluminosilicate components used to manufacture the binder. Depending on the location and technology of the industrial enterprise, the waste it receives in the form of slag and ash can vary greatly in chemical composition, which makes the developed recipe limited, and its practical use with aluminosilicate components of other origins may not be as effective and will require additional adjustment of the composition of geopolymer concrete.

## 4. Conclusions

Several types of scientific proposals for improving the properties of geopolymer concrete have been studied and tested. The optimal combination of aluminosilicate binders for the production of geopolymer concrete has been selected. The optimal content of polypropylene fiber for dispersed reinforcement of geopolymer concrete has been determined.

(1) It has been established that with an increase in the FA content, the cone slump decreases. The difference in the slump between the GGBS-based composition and the FA-based composition was 53.3%. FA particles are smaller and have a larger specific surface area compared to GGBS particles, and therefore, more water is required for their wetting, which leads to a decrease in the slump of the mixtures.

(2) The optimal composition of the geopolymer binder with a content of 80% GGBS and 20% FA has been determined. This combination of aluminosilicate components made it possible to obtain geopolymer concrete with a maximum compressive strength of 31.5 MPa. With this combination of GGBS and FA, the polymerization reactions proceed most actively with a large number of CSHs.

(3) Dispersed PF reinforcement improves the strength properties of geopolymer concrete. The PF content in the composite 0.8% is the most optimal. With a content of 0.8% PF, the maximum values of compressive and flexural strength were recorded, the increases which amounted to 7.3% and 48.7%, respectively. At the optimal dosage, polypropylene fibers are uniformly distributed throughout the structure of geopolymer concrete and create additional bonds, which subsequently lead to the strengthening of the entire structure of the composite.

(4) Electron and optical microscopy analysis of the geopolymer matrix based on a mixture of 80% GGBS and 20% FA showed a well-compacted microstructure and a large number of gel-like products of the geopolymerization reaction. No unreacted GGBS and FA particles were recorded. The PF at the fiber–matrix boundary is torn out of the matrix structure and deformed under the action of destructive loads. The nature of the destruction at the fiber–matrix boundary indicates a fairly good adhesion of the matrix and fiber.

(5) The resulting geopolymer fiber concrete on a combined binder has a compressive strength of 33.8 MPa and a flexural strength of 5.8 MPa, which determines its wide range of applications in real construction practice.

Further research is planned in the direction of studying the stress–strain behavior of geopolymer concrete using polymer fibers, as well as durability properties such as frost resistance, sulfate and chloride resistance and water resistance.

## Figures and Tables

**Table 1:** Chemical composition of GGBS and FA.

Oxid	GGBS	FA
SiO_2_ (%)	31.1	50.82
Al_2_O_3_ (%)	7.09	20.5
Fe_2_O_3_ (%)	0.51	7.71
CaO (%)	38.5	5.55
MgO (%)	4.32	2.18
TiO_2_ (%)	0.28	0.87
Р_2_О_5_ (%)	0.03	0.21
SO_3_ (%)	2.19	0.16
LOI (%)	15.98	12.0
Bulk density (kg/m^3^)	1098	1013

**Table 2:** Compositions of geopolymer concrete on a combined binder.

Mixture Type	GGBS/FA (%)	GGBS (kg/m^3^)	FA (kg/m^3^)	QS (kg/m^3^)	CrS (kg/m^3^)	NaOH (kg/m)^3^	Water (L/m^3^)
100G/0F	100/0	488	0	485	1272	31	190
80G/20F	80/20	390.4	97.6	485	1272	31	190
60G/40F	60/40	292.8	195.2	485	1272	31	190
40G/60F	40/60	195.2	292.8	485	1272	31	190
20G/80F	20/80	97.6	390.4	485	1272	31	190
0G/100F	0/100	0	488	485	1272	31	190

**Table 3:** Compositions of geopolymer fiber concrete on a combined binder.

Mixture Type	GGBS (kg/m^3^)	FA (kg/m^3^)	QS (kg/m^3^)	CrS (kg/m^3^)	PF (kg/m)^3^	NaOH (kg/m)^3^	Water (L/m^3^)
PF/0.2	390.4	97.6	485	1272	0.98	31	190
PF/0.4	390.4	97.6	485	1272	1.95	31	190
PF/0.6	390.4	97.6	485	1272	2.93	31	190
PF/0.8	390.4	97.6	485	1272	3.90	31	190
PF/1.0	390.4	97.6	485	1272	4.88	31	190
PF/1.2	390.4	97.6	485	1272	5.86	31	190

**Table 4:** Processing of the results obtained during the tests.

Name of the Indicator	Calculation Formula
Cone slump	Difference between the height of the mold and the height of the highest point of the settled geopolymer mixture. Average value based on the results of two tests.
Mixture density	$\rho_{fc}=\frac{m-m_{1}}{V}\times 1000$ (1) here *m* is the mass of the measuring vessel with concrete mixture (g); *m*_1_ is the mass of the measuring vessel without mixture (g); *V* is the capacity of the measuring vessel (cm^3^).
Concrete density	$\rho =\frac{m}{V}\times 1000$ (2) here *m* is the mass of the sample (g); *V* is the volume (cm^3^)
Compressive strength	$R=\alpha \frac{F}{A}$ (3) here *F* is the breaking load (N); *A* is the area of the working cross-section of the sample (mm^2^); *α* is the coefficient considering the dimensions of the samples (for samples with a side of 100 mm α = 0.95).
Bending strength	$R_{bt}=\delta \frac{F\;l}{a\;b^{2}}$ (4) here *F* is the breaking load (N); *a*, *b*, *l* are the width, height of the prism cross-section and the distance between the supports, respectively, when testing samples for tensile strength under bending (mm); *δ* is the coefficient considering the dimensions of the samples (for samples with a side of 100 mm *δ* = 0.92).

**Table 5:** Analysis of the effect of combined geopolymer binders on the properties of the geopolymer composite.

Ref Number	Type of Aluminosilicate Component	Proportion	The Obtained Result
[71]	Slag/microsilica/metakaolin	70%/20%/10%	Repair geopolymer composition with good adhesive properties
[72]	Fly ash/slag	50%/50%	Geopolymer concrete with high resistance to chloride corrosion
[73]		70%/30%	
[52]	Slag/fly ash/limestone	60%/40%/+5%	Geopolymer composite with compressive strength of 42 MPa
[53]	Diabase sludge/slag	30%/70%	Geopolymer composite with reduced shrinkage and compressive strength of 98.05 MPa
[74]	Slag/oyster shell powder	90%/10%	Efficient and environmentally friendly composites with the required performance properties and improved durability properties
[75]	Slag/concrete and brick waste powder	45%/55%	

**Table 6:** Analysis of the effect of dispersed reinforcement with polypropylene fiber on the properties of geopolymer concrete.

Ref Number	FIBER TYPE	Optimum Content	Obtained Result
[79]	Glass/Basalt	1%/1%	Hinged geopolymer beams with the addition of glass and basalt fiber showed strength of 49% and 37% higher than that of the control geopolymer concrete
[80]	Polypropylene	0.2–0.4%	Geopolymer solutions with improved strength and reduced permeability were obtained
[81]	Steel	3%	Inclusion of steel fiber in geopolymer concrete after 28 days allowed to obtain a composite with a compressive strength of 107 MPa
[82]		0.3%	Increase in compressive strength of permeable geopolymer composite up to 43.62%
[83]	Recycled fiberglass	2–6%	Compressive strength of foamed geopolymer composite increased by 25–165%, and shrinkage decreased by 55%
[84]	Mullite fiber	10%	Increase in compressive strength up to 20%
[85]	Basalt	1–2%	Geopolymer composites with improved strength and performance properties were obtained
[86]			
[87,88,89]	Polypropylene	0.25–2%	

## Data Availability

The original contributions presented in the study are included in the article; further inquiries can be directed at the corresponding author.
